# The Treatment of Cartilage Damage Using Human Mesenchymal Stem Cell-Derived Extracellular Vesicles: A Systematic Review of *in vivo* Studies

**DOI:** 10.3389/fbioe.2020.00580

**Published:** 2020-06-11

**Authors:** Kendrick To, Karl Romain, Christopher Mak, Achi Kamaraj, Frances Henson, Wasim Khan

**Affiliations:** ^1^Division of Trauma and Orthopaedics, Department of Surgery, University of Cambridge, Cambridge, United Kingdom; ^2^School of Clinical Medicine, University of Cambridge, Cambridge, United Kingdom

**Keywords:** extracellular vesicle, mesenchymal stem cell, cartilage, tissue engineering, osteoarthritis

## Abstract

Damage to joints through injury or disease can result in cartilage loss, which if left untreated can lead to inflammation and ultimately osteoarthritis. There is currently no cure for osteoarthritis and management focusses on symptom control. End-stage osteoarthritis can be debilitating and ultimately requires joint replacement in order to maintain function. Therefore, there is growing interest in innovative therapies for cartilage repair. In this systematic literature review, we sought to explore the *in vivo* evidence for the use of human Mesenchymal Stem Cell-derived Extracellular Vesicles (MSC-EVs) for treating cartilage damage. We conducted a systematic literature review in accordance with the PRISMA protocol on the evidence for the treatment of cartilage damage using human MSC-EVs. Studies examining *in vivo* models of cartilage damage were included. A risk of bias analysis of the studies was conducted using the SYRCLE tool. Ten case-control studies were identified in our review, including a total of 159 murine subjects. MSC-EVs were harvested from a variety of human tissues. Five studies induced osteoarthritis, including cartilage loss through surgical joint destabilization, two studies directly created osteochondral lesions and three studies used collagenase to cause cartilage loss. All studies in this review reported reduced cartilage loss following treatment with MSC-EVs, and without significant complications. We conclude that transplantation of MSC-derived EVs into damaged cartilage can effectively reduce cartilage loss in murine models of cartilage injury. Additional randomized studies in animal models that recapitulates human osteoarthritis will be necessary in order to establish findings that inform clinical safety in humans.

## Introduction

Damage to joints through injury or disease can result in cartilage loss, which if left untreated can lead to inflammation and ultimately osteoarthritis (OA) (Davies-Tuck et al., 2008). OA affects up to three out of 10 people over the age of 60 years (Woolf and Pfleger, 2003), and this is projected to increase substantially (Turkiewicz et al., 2014). There is currently no cure for OA and management is focused on symptom control (Mcalindon et al., 2014). Furthermore, the search for Disease Modifying Osteoarthritis Drugs (DMOAD) has not been fruitful, and there are no approved DMOADs. End-stage OA can be severely debilitating and ultimately requires joint replacement in order to maintain function (Gillam et al., 2013). Joint replacement is costly and carries perioperative morbidity (Berstock et al., 2014) as well as unsatisfactory outcomes (Nilsdotter et al., 2003). Therefore, there is a need for innovative therapies to treat cartilage defects and in doing so, prevent OA.

The established treatment of microfracture for focal cartilage defects aims to encourage endogenous cells to repopulate areas of cartilage loss, but this has demonstrated limited effectiveness (Weber et al., 2018). A large number of studies have investigated tissue engineering and cellular regenerative approaches to treating cartilage defects (Negoro et al., 2018). Acellular biomaterial scaffolds are costly to develop and implantation of these scaffolds into cartilage defects exhibits a high failure rate (Vindas Bolaños et al., 2017). Certain cell-based approaches such as Autologous Chondrocyte Implantation (ACI) can be effective but cause donor-site morbidity (Reddy et al., 2007; Bexkens et al., 2017). Recently, there has been an increasing body of evidence to support the use of Mesenchymal Stem Cells (MSCs) in cartilage repair (Borakati et al., 2018).

MSCs are multipotent adult stromal cells that may be derived from a number of tissues including bone marrow, synovium, adipose, umbilical cord and dental pulp, and so are readily available for autologous harvest (Fernandes et al., 2018; Fabre et al., 2019). Ease of extraction and the potential for *ex vivo* expansion make MSCs an attractive option for tissue repair. To this end, studies have shown the therapeutic potential of MSC transplantation in promoting regeneration of tissues such as bone, cartilage, and nerve (Katagiri et al., 2017; Freitag et al., 2019; Masgutov et al., 2019). However, MSC transplantation is not without risks. Certain studies have revealed potential immunogenic complications related to repeated allogenic transplantation of MSCs (Cho et al., 2008) and others have reported possible tumorigenic properties (Beckermann et al., 2008). There is also *in vivo* evidence to suggest that, when transplanted in the presence of malignancy, MSCs may increase the risk of metastasis (Karnoub et al., 2007). Suboptimal engraftment and delocalization from the target site create difficulty in maintaining sustained benefit following transplantation, and suggest that observed long-term benefits may not result from MSC differentiation alone (Zwolanek et al., 2017).

Increasingly so, studies are focussing on the paracrine function of MSCs as the predominant mechanism of their regenerative effects (Linero and Chaparro, 2014; Xu et al., 2016). MSC-derived extracellular vesicles (MSC-EVs) are gaining interest as a cell-free therapeutic option for cartilage repair. As part of MSC secretome, EVs are nanovesicles ranging from 10 nm to several micrometers that contain various components including genetic material in the form of messenger RNA (mRNA), microRNA (miRNA), lipids and bioactive proteins (Di Vizio et al., 2012; Huang et al., 2017; Théry et al., 2018). MSC-EVs are characterized by cell-surface expression of generic EV markers such as CD9, CD81, CD82, TSG101, and Alix. Transplantation of MSC-EVs may carry certain advantages over cell-based therapies. Firstly, accurate quantification of number of transplanted MSCs may be difficult and their effects could also be less predictable than that of EVs within the recipient site, potentially making outcomes of clinical trials less reproducible. Production costs of EVs at a large scale could be lower than that of MSCs (Cha et al., 2018). Furthermore, EVs exert low potential for toxicity and immunogenicity with repeated transplantation (Zhu X. et al., 2017; Saleh et al., 2019), and therefore avoid the undesired immunogenic properties of MSCs (Gu et al., 2015). As a cell-free therapy, MSC-EVs can be stored by cryopreservation whereas MSCs cannot, and therefore have greater potential as an off-the-shelf treatment option (Vlassov et al., 2012).

Through mechanisms including direct receptor interaction, membrane fusion, and internalization, EVs are able to influence recipient cell behavior to promote a variety of effects relevant to cartilage repair. *In vitro* evidence shows that MSC-EVs exert anti-inflammatory effects through influencing IL-6 and TGF-β secretion by dendritic cells. Furthermore, MSC-EVs are found to contain miRNAs such as miR-21-5p which target the CCR7 gene for degradation (Reis et al., 2018) and non-coding RNA that mediate an anti-inflammatory response (Fatima et al., 2017). Co-culture of MSCs with chondrocytes is found to promote matrix production and chondrocyte chondrogenesis *in vitro*, and these effects appear to be EV-dependent (Kim et al., 2019). *In vitro* studies also suggest that chondrocytes take-up MSC-EVs that upregulate type II collagen production (Vonk et al., 2018). Finally, results of recent *ex vivo* studies have suggested that MSC-conditioned media is able to downregulate the expression of genes that promote extra-cellular matrix degradation such as MMP1, MMP13, and IL-1β in synovial explants (van Buul et al., 2012) facilitating cartilage repair, suggesting a role for EVs (Nawaz et al., 2018).

Recently, there has been increasing interest in the use of MSC-EVs in cartilage repair and some systematic reviews have examined the *in vitro* evidence for animal MSC-EVs. In this systematic literature review, we sought to explore the *in vivo* evidence for the use of human-derived MSC-EVs in murine models of cartilage repair.

## Materials and Methods

**Figure 1:**
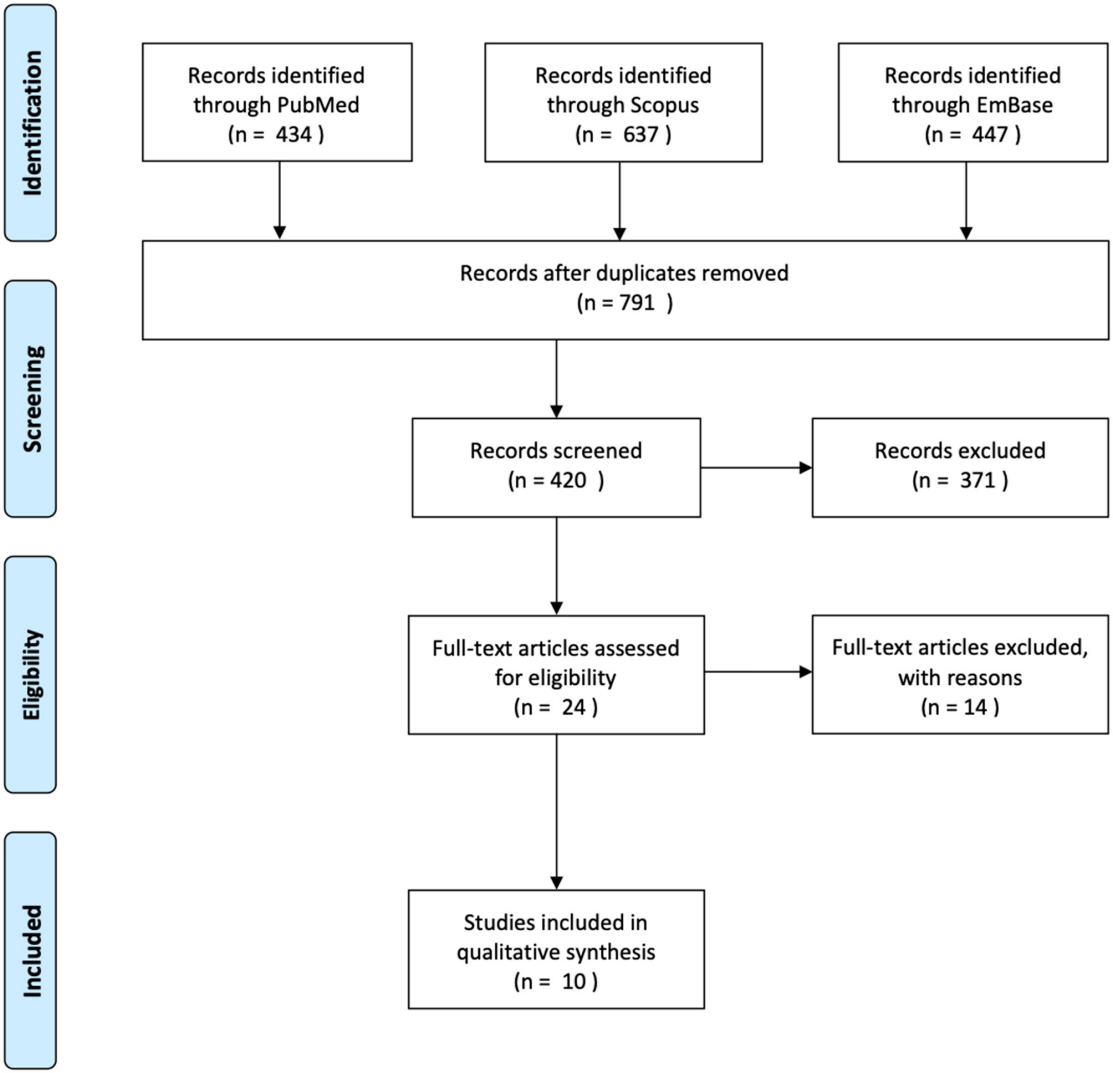
Prisma flow diagram.

The methods used to conduct this review were according to the Preferred Reporting Items for Systematic Reviews and Meta-Analyses (PRISMA) statement protocol (Moher et al., 2015). We carried out a literature search on PubMed, Scopus, and EmBase databases in January 2020, capturing articles starting from conception. The following search strategy was applied: (Mesenchymal stem cell OR MSC^*^ OR Multipotent stromal cell OR Multipotent stem cell OR Mesenchymal stromal cell) AND (Extra-cellular vesicle OR extracellular vesicle OR EV^*^ OR exosomal OR exosome) AND (osteoarthritis OR OA^*^ OR osteochondral OR Cartilage). Following de-duplication, exclusion criteria was applied to studies not written in or translated into the English language. We excluded studies that only performed *in vitro* experiments. Studies that did not characterize or validate the cell populations as per the recommendations of the International Society for Cellular Therapy (ISCT) for MSC were excluded (Witwer et al., 2019). We included studies that examined the effects of human MSC-derived exosomes, studies examining animal MSC-derived exosomes were excluded. Studies that conducted characterization of EVs in accordance with The International Society for Extracellular Vesicles (ISEV) standards were included (Théry et al., 2018). We included case-control studies, randomized control trials, case series, and case reports with two or more subjects. After removing duplicates, a total of 727 studies underwent title screening (Figure 1). A total of 24 studies were examined in full text. Ten studies were included in our review.

Quality assessment was carried out independently KR and CM using the SYstematic Review Center for Laboratory animal Experimentation (SYRCLE) tool (Hooijmans et al., 2014), discrepancies in results were resolved by discussion.

## Results

### MSC Characteristics

We identified 10 studies in our review, all of which were case-control studies with murine subjects. MSCs were derived from a variety of human tissue sources (Table 1). Four studies (Wang et al., 2017), three by the same group, used MSCs derived from embryonic stem cells (Zhang et al., 2016, 2018, 2019). Three studies obtained MSCs from bone marrow aspirate (Khatab et al., 2018; Mao et al., 2018; Jin et al., 2020), one from infrapatellar fat pad (Wu et al., 2019) and one from synovial tissue (Tao et al., 2017). One study compared EVs from Induced Pluripotent Stem Cell (iPS)-derived MSCs with synovium-derived MSCs (Zhu Y. et al., 2017). All MSCs were characterized using flow cytometry and trilineage differentiation. All MSCs expressed either CD44, CD90 or CD105, and most expressed low levels of HLA-DR.

**Table 1:** Method of MSC harvest and characterization.

**References**	**Source**	**Cell harvest**	**Cell treatment**	**MSC characterization**
Khatab et al. (2018)	Human	Heparinised femoral shaft bone marrow aspirate	Cultured in minimal essential medium alpha (α MEM), Fetal Calf Serum (FCS) and Invitrogen to third passage	Trilineage differentiation, Flow cytometry: CD73, CD90, CD105, CD166 +ve
Mao et al. (2018)	Human	Bone marrow aspirate from iliac crest	Cultured in ^a^standard Mesenchymal Stem Cell (MSC) media and changed to chondrogenic media from third passage onwards. miR-92a-3p overexpressed in one group	Trilineage differentiation, Flow cytometry: CD11b, CD19, CD34, CD45, CD73, CD90, CD105, and HLA-DR –ve
Tao et al. (2017)	Human	Synovial membrane tissue	Cultured in standard MSC media to fifth passage. miR-140-5p overexpressed in one group	Trilineage differentiation, Flow cytometry: CD44, CD73, CD90, CD105, CD151 +ve
Wang et al. (2017)	Human	Embryonic stem cell-derived MSCs (obtained from third party)	Cultured in standard MSC media and cells between fourth and seventh passage were utilized	Trilineage differentiation, Flow cytometry: CD73, CD90, CD105 +ve
Wu et al. (2019)	Human	Infrapatellar fat pad obtained following total knee arthroplasty	Cultured in standard MSC media to confluence and used at first passage	Flow Cytometry: CD44, CD73, CD90 +ve. CD34, CD11b, CD19, CD45, HLA-DR present at low levels
Zhang et al. (2016)	Human	Cleavage and blastocyst- stage embryonic stem cells from *in vitro* fertilization	Cultured in standard MSC media. Further details not stated	Trilineage differentiation, Flow cytometry: CD105, CD24 +ve
Zhang et al. (2018)	Human	Immortalized E1-Myc 16.3 embryonic stem cell-derived MSC	Cultured in standard MSC media and passaged at 80% confluence until use. Grown in defined media for 3 days prior to exosome extraction	Trilineage differentiation, Flow cytometry: CD29, CD44, CD90, CD105 +ve, CD34, CD45, HLA-DR –ve
Zhang et al. (2019)	Human	Immortalized E1-Myc 16.3 embryonic stem cell-derived MSC	Cultured in standard MSC media and passaged at 80% confluence until use. Grown in defined media for 3 days prior to exosome extraction	Trilineage differentiation, Flow cytometry: CD29, CD44, CD90, CD105 +ve, CD34, CD45, HLA-DR –ve
Zhu Y. et al. (2017)	Human	Induced pluripotent stem cell-derived MSC (iMSC) induced from human umbilical cord iPS Synovial MSC (sMSC) from humans undergoing Anterior crucial efforts ligament (ACL) reconstruction	iMSC: iPS cultured for 5 days in mTESR1 (Stemcell) and then cultured in standard MSC media for 2 weeks. The cells were then passaged every 5–7 days until a fibroblastic morphology was adopted sMSC: cultured in standard MSC media, with media changed every 4 days	Trilineage differentiation Flow cytometry: iMSC: CD29, CD44, CD73, CD90 +ve. CD34, CD45, HLA-DR –ve sMSC: CD44, CD73, CD90, CD166 +ve. CD34, CD4, HLA-DR –ve
Jin et al. (2020)	Human	MSCs derived from bone marrow aspirate from the ilium of healthy subjects	Cultured to third to fifth passage with media changed every 48 h	Trilineage differentiation. Flow cytometry: CD29, CD44, CD71 +ve. CD34, CD45, and HLA-DR -ve

a *refers to individual culture condition protocol without addition of stimulating factors*.

### EV Characteristics

Ultracentrifugation and ultrafiltration were the two most common methods for isolation of exosomes (Table 2). One study used polyethylene glycol precipitation as part of the purification process (Wu et al., 2019), and several used tangential flow filtration (TFF). EV dimensions were determined using Transmission Electron Microscopy (TEM) in all but one study (Khatab et al., 2018). EV size ranged from 30 to 200 nm, with a modal mean size of around 100 nm. Flow cytometry and western blotting were standard methods for characterizing EVs. CD9, CD63, CD81, and ALIX were the most common EV markers identified. Five out of 10 studies that determined the bioactive component of the EVs used with Reverse Transcriptase quantitative Polymerase Chain Reaction (RT-qPCR) or Western blot analysis. Three studies attributed the effects of EVs to various miRNA. Two studies determined that CD73-mediated protein kinase activation was responsible for the *in vivo* effects of EV transplantation (Zhang et al., 2018, 2019).

**Table 2:** Method of EV purification and characterization.

**References**	**Purification process**	**EV dimensions**	**EV marker**	**Imaging**	**Active component**
Khatab et al. (2018)	Ultracentrifugation	Not determined	Not determined	Not utilized	Not determined
Mao et al. (2018)	Ultracentrifugation	50–150 nm	CD9, CD63, CD81, HSP70	Transmission electron microscopy (TEM)	miR-92a-3p
Tao et al. (2017)	Not specified	30–150 nm	CD63, CD9, CD81, ALIX	TEM, DLS	Not determined
Wang et al. (2017)	Ultracentrifugation	30–200 nm	CD63, CD9	TEM	Not determined
Wu et al. (2019)	Ultrafiltration and polyethylene glycol precipitation	30–150 nm, main peak at 125.9 nm	CD9, CD63, CD81	TEM	miR-199-3p, miR-99-5p, MiR-100-5p (targeting 3'UTR of mTOR)
Zhang et al. (2016)	Culture media concentrated by Tangential Flow Filtration (TFF) sequentially through membranes (1,000 kDa, 500 kDa, 300 kDa, and 100 kDa) then filtered through 0.2 um filter	Homogenously sized particles; modal size of 100 nm	CD81, TSG101, ALIX	TEM	Not determined
Zhang et al. (2018)	Conditioned medium was size fractionated and concentrated by TFF	Homogenously sized particles; modal size of 100 nm	CD81, TSG101, ALIX	TEM	CD73-mediated adenosine activation of MAPK signaling
Zhang et al. (2019)	Conditioned medium was sized fractionated and concentrated by TFF	Particles between 100 and 200 nm	CD81, TSG101, ALIX	TEM	CD73 mediated activation of MAPK signaling
Zhu X. et al. (2017)	Conditioned media concentrated by centrifugation and ultrafiltration	Tunable Resistive Pulse Sensing (TRPS): 50–150 nm TEM: 50–200 nm	CD9, CD63, TSG101	TEM, TRPS	Not determined
Jin et al. (2020)	Conditioned media extracted using size fractionation and filtration	50–100 nm	CD63, CD9, Hsp70	TEM	miR-26a-5p

### Animal Models

All studies used murine models; five studies induced osteoarthritis, including cartilage loss through surgical joint destabilization, two studies directly created osteochondral lesions and three studies used collagenase to cause cartilage loss. All but one study, which examined the TMJ, studied the knee joint. Follow-up duration ranged from 3 to 12 weeks after induction of cartilage injury. All studies delivered EVs via intra-articular injection. No significant side-effects were reported in any subjects. Varying amounts of EVs were used between studies, and the amount was quantified using different measures (Table 3). Some studies injected a given volume with a known concentration of EVs, whereas others determined the mass of EVs delivered.

**Table 3:** Summary of findings from *in vivo* experiments.

**References**	**Method of delivery**	**Injury model**	**Duration of follow up**	**Macroscopic appearance/functional studies**	**Imaging and histology**	**Biochemical analysis**
Khatab et al. (2018)	Intra-articular injection of secretome (Derived from 20,000 third passage MSCs suspended in 6 μl medium) on day 7, 9, and 11 after OA induction	Murine, Collagenase Induced Osteoarthritis (CIOA)	21 days	Greater pain reduction in OA-affected limb from day 7 in treated groups	Histological assessment revealed improved cartilage thickness but no treatment effect on subchondral bone volume	Immunostaining of iNOS, CD163, and CD206 did not demonstrate a difference between treated and untreated groups
Mao et al. (2018)	Intra-articular injection of 15 μl of MSC-derived exosomes or MSC-derived exosomes from a group pre-treated with miR-92a-3p-Exos On days 7, 14, and 21	Murine, CIOA	28 days	Improved cartilage appearance in treated groups	Improved microscopic cartilage matrix appearance	Greater COL2a1 and aggrecan staining in treated lesions. Increased regulation of WNT5A, COL2A1, and aggrecan mRNA expression
Tao et al. (2017)	Intra-articular injection of 100 μl of 10^11^ exosome particles/mL weekly from week 5 to 8 post-surgery	Murine, medial meniscus, and medial collateral ligament transection	12 weeks	Not undertaken	Histology: Less joint wear and cartilage matrix loss in the treated group undertaken. Improved Osteoarthritis Research Society International (OARSI) score	Immunostaining: greater type II collagen (Col II), aggrecan, and type I collagen expression
Wang et al. (2017)	Intra-articular injection of 5 μl of exosomes into Knee joint at week 4 and every 3 days thereafter	Murine, destabilization of medial meniscus (DMM)	8 weeks	Not undertaken	Improved OARSI score in treated group. Reduced microscopic appearance of OA in treated group	Greater Col II staining and weaker ADAMTS5 staining in the treated group
Wu et al. (2019)	Intra-articular injection of 10 μl of exosome (10^10^ particles/ml) weekly or biweekly	Murine, DMM	8 weeks	Improved gait; increased weight bearing on OA knee, swing speed and intensity in treated groups	Improved OARSI score in the treated group	Immunohistology showed increased Col II expression, decreased ADAMTS5 and MMP13 expression.
Zhang et al. (2016)	Intra-articular injection of 100 μg of exosomes weekly from surgery	Murine, surgically induced osteochondral defects on trochlear grooves of distal femur	12 weeks	Macroscopic: Moderate improvement at 6 weeks, near-complete neotissue coverage and integration with surrounding cartilage at 12 weeks in treated groups. Improved International Cartilage Repair Society (ICRS) score at 12 vs. 6 weeks	Histology: smooth cartilage in five out of six treated defects at 12 weeks	Immunohistochemistry: intense GAG staining (>80%), high level of T2Col and low level of T1Col in treated lesions. Lubricin +ve cells found in superficial and middle zones of neo-cartilage
Zhang et al. (2018)	Intra-articular injection of 100 μg of exosomes weekly from surgery	Murine, Surgically induced osteochondral defects on trochlear grooves of distal femur	12 Weeks	Improved Wakitani macroscopic score at 2, 6, and 12 weeks compared to controls	More neotissue formation compared to control	Increased GAG and T2Col staining in treated groups. Increased Proliferative Cell Nuclear Antigen (PCNA) and decreased Cleaved Caspase-3 (CCP3) at 12 weeks in treated groups
Zhang et al. (2019)	Intra-articular injection of 100 μg of exosomes at weekly intervals starting from 2 weeks following OA induction	Murine, monosodium iodoacetate (MIA) induced cartilage loss in temporomandibular joint (TMJ) by inject of Monosodium iodoacetate (MIA) into upper compartment of the joint bilaterally	12 weeks	Reduced Pain behavior in exosome treated rats from 2 weeks onwards as indicated by higher Head Withdrawal Threshold (HWT) as stimulated by Von Frey fibers	Micro-CT: Improved condylar height, cartilage thickness, matrix deposition and subchondral bone integrity from 8 weeks post treatment Histology: Improved Mankin score in treated groups at 4 weeks onwards	Immunohistochemistry: Increased GAG and T2Col staining in treated groups. Increased Proliferative Cell Nuclear Antigen (PCNA) and decreased Cleaved Caspase-3 (CCP3) at 12 weeks in treated groups. PCR: reduced expression of IL-1B, BAX, alpha-SMA) and Substance P, Nerve Growth Factor (NGF), Tyrosine receptor Kinase A (TrkA). Increased TIMP2 expression. Decreased ADAMTS5 expression
Zhu X. et al. (2017)	Intra-articular injection with 8 μl (10^10^/ml) of exosomes on day 7,14, and 21 following OA induction	Murine, CIOA	4 weeks	Improved ICRS score in both iMSC and sMSC group compared with OA group at endpoint	Improved OARSI score in all groups compared to untreated control. iMSC showed a greater improvement in OARSI score than sMSC	Greater Col II staining in treated groups. Greater Col II staining in iMSC compared to sMSC groups
Jin et al. (2020)	Intra-articular injection of 250 ng of exosomes in 5 μl	Murine, anterior crucial ligament, posterior cruciate ligament, medial cruciate ligament, lateral cruciate ligament, medial and lateral meniscus transection	8 weeks	Not undertaken	Improved microscopic appearance, greater synovial cell infiltration and fibrous tissue formation in treated groups	Reduced MMP-3 and MMP-13 expression in treated group. Reduced synovial cell apoptosis in treated group. Decreased IL-1B in treated groups. Downregulation of PTGS2

### *In vivo* Findings

Two studies measured pain scores and one conducted gait analysis following treatment with EVs, and all three showed improved functional scores. Zhang et al. (2019) used Micro-Computed Tomography (micro-CT) to assess cartilage morphology and found improved bone integrity from 8 weeks onwards. Gene-expression analysis undertaken in several studies. Mao et al. reported increased chondrogenic gene regulation after EV treatment. Using PCR, Zhang et al. (2019) and Jin et al. detected reduced regulation of pro-inflammatory cytokines in their studies.

Joint appearance was interrogated macroscopically, microscopically or using both approaches. All studies reported a reduction in cartilage loss after treatment with EVs. Microscopic histological analysis focused on assessing cartilage thickness and cartilage matrix appearance. Four studies conducted subjective quantification of appearance using the OARSI score. All studies found improved cartilage appearance or OARSI score in the treated groups, although Khatab et al. reported no treatment effect on subchondral bone volume at 3 weeks. Immunohistology was utilized in all studies and all reported increased collagen type II staining with several groups reporting reduced MMP3 immunostaining. Three studies reported reduced staining of apoptotic markers.

### Quality of Studies

The SYRCLE tool was used to grade each study using 15 different parameters. Seven out of the 10 studies had a low level of concern overall and three studies had some concern toward risk of bias (Table 4). Blinding and detection bias constituted the main contributors to bias in the studies. There was little selection and reporting bias among the studies, but randomization of subjects were not mentioned in most studies. Overall, the studies included in this review were of high quality and low risk of bias.

**Table 4:** Summary of risk of bias analysis.

**References**		**Selection bias in sequence generation**	**Selection bias in baseline characteristics**	**Selection bias in allocation concealment**	**Performance bias in random housing**	**Performance bias in blinding**	**Detected bias in random outcome assessment**	**Detected bias in blinding**	**Attrition bias in incomplete outcome data**	**Reportingbias**	**Number of Subjects (*n* =)**	**Number of Controls (*n* =)**	**Selectionbias**	**Performancebias**	**Detectionbias**	**Attritionbias**	**Reportingbias**	**Overall**
Khatab et al. (2018)	Mice	No	No	No	No	Unclear	Unclear	No	No	No	11	11	No	No	No	No	No	No
Mao et al. (2018)	Mice	No	No	No	Unclear	Unclear	Unclear	yes	No	No	10	10	No	Unclear	yes	No	No	Some concerns
Tao et al. (2017)	rat	No	No	No	No	Unclear	Unclear	No	No	No	10	10	No	No	No	No	No	No
Wang et al. (2017)	Mice	No	No	No	Unclear	Unclear	Unclear	No	No	No	20	12	No	Unclear	No	No	No	No
Wu et al. (2019)	Mice	No	No	No	Unclear	Unclear	Unclear	yes	No	No	8	8	No	Unclear	yes	No	No	Some concerns
Zhang et al. (2016)	rat	No	No	No	Unclear	Unclear	Unclear	No	No	No	12	12	No	Unclear	No	No	No	No
Zhang et al. (2018)	rat	No	No	No	Unclear	Unclear	Unclear	No	No	No	36	36	No	Unclear	No	No	No	No
Zhang et al. (2019)	rat	No	No	No	Unclear	Unclear	Unclear	yes	No	No	32	14	No	Unclear	yes	No	No	Some concerns
Zhu X. et al. (2017)	Mice	No	No	No	Unclear	Unclear	Unclear	No	No	No	10	5	No	Unclear	No	No	No	No
Jin et al. (2020)	Mice	No	No	No	Unclear	Unclear	Unclear	No	No	No	10	10	No	Unclear	No	No	No	No

## Discussion

All 10 studies in this review reported reduced cartilage loss following treatment with MSC-EVs. A variety of outcome measures were employed in each study to examine the impact of EVs on cartilage loss, but not all studies found improvements in every parameter measured. A total of 159 subjects were treated with MSC-EVs without significant or immunogenic complications. While all the MSC-EVs were derived from human MSCs, the subjects were all animal models of cartilage injury, and therefore only indirectly inform safety and effectiveness in human subjects.

The capacity for MSCs to expand *ex vivo* varies with cell source (Fazzina et al., 2016) and anatomical site (Davies et al., 2017). The ability of MSCs to undergo chondrogenic differentiation also appears to differ between cell source (Bernardo et al., 2007). The source of EVs varied significantly between studies as the MSCs were harvested from different tissues and anatomical donor sites. The influence of MSC source on the chondrogenic potential of EVs remains unclear, with some studies suggesting that certain MSC-EVs reduce type I and III collagen production (Li et al., 2013). There is evidence that the biological properties of EVs are dependent on MSC source (Kehl et al., 2019) and it is also apparent that certain MSCs, for example those derived from amniotic fluid, may produce a greater number of EVs than bone marrow-derived MSCs when controlled for cell number (Tracy et al., 2019). In this review, two studies used MSCs from immortalized cell lines (Zhang et al., 2018, 2019) this provides an advantage over autologous harvest as it is not invasive. Zhu et al. compared iPS-derived MSC-EVs with sMSC-derived EVs and found the former to be superior in cartilage repair. Relative cost and convenience of production will dictate which of these is favorable. These are important considerations in tissue engineering as the optimal source cell should achieve a balance between ease of harvest and acceptable EV production. MSC-EV bioactivity also appear to depend on cell-culture conditions, for example, the anti-apoptotic effects of adipose-derived MSC secretome can be affected by oxygen tension (An et al., 2015). Therefore, future studies will be required to delineate the relationship between MSC cell source and secretome in order to select the best source for optimal large-scale EV production.

Most studies in the literature examining EV function, in line with our findings, use ultracentrifugation as the main component of the isolation procedure (Gardiner et al., 2016). There is evidence that suggests that ultracentrifugation could increase the amount of contamination by macromolecules within the MSC culture media (Webber and Clayton, 2013). This may be of relevance in our interpretation as MSC-conditioned media is known to contain non-vesicular bioactive components that may promote chondrogenesis (Chen et al., 2018) and lead to an overestimation of EV effectiveness in cartilage repair. While this may make comparisons difficult, the augmented repair is not necessarily an undesired effect. Furthermore, while the effect of multiple washing stages that form part of the centrifugation process improves purity, it may decrease the total number of EVs obtained (Webber and Clayton, 2013) TFF was the next most commonly used method of concentrating EVs. Compared to ultracentrifugation, TFF achieves a greater EV yield, with a reduced amount of non-vesicular macromolecules contamination (Busatto et al., 2018). Other forms of flow-based purification such as Cross-flow isolation are also favorable over ultracentrifugation in terms of rate of production at large scale (McNamara et al., 2018). Ultimately, robust cost-effectiveness studies may be required to determine the optimal method of purification. The transferability of this review may also be limited by the inconsistent methodologies used by the studies to characterize the EVs used. Apart from one study that did not report on any EV markers (Khatab et al., 2018), all other studies identified markers recognized by the Minimal Information for Studies of Extracellular Vesicles (MISEV) criteria (Théry et al., 2018). EV dimensions were found to range from 30 to 200 nm. The range of 50–150 nm was most commonly reported and may reflect the bias of measurement devices. Future studies should attempt to ascertain the main peak value within the detected range as Wu et al. (2019) did in their study.

Whilst all studies directly injected EVs intra-articularly, the dose delivered was highly varied. Although EV transplantation is yet to be tested in clinical studies, proof-of-concept human clinical trials of intra-articular injection of MSCs to treat cartilage lesions demonstrate a dose-dependent effect without increased risk of adverse effects (Jo et al., 2014). Similar studies will be required to establish such a relationship for EV transplantation. In MSC treatment of cartilage lesions, intra-articular injection appear to promote good engraftment rates with minimal off-site engraftment (Satué et al., 2019). In *in vivo* studies of anterior cruciate ligament repair, intravenous injection of MSCs concomitantly with intra-articular injection produced improved outcomes (Muir et al., 2016). Likewise, intravenous EV injection appear to produce a dose-dependent immunosuppressive effect that may be beneficial for treating arthritis (Cosenza et al., 2018), but the effect on cartilage repair remains unknown, and the potential for off-site engraftment of EVs is not yet characterized.

All animal studies to date on human-derived MSC-EVs have focussed on murine models. Articular cartilage repair can be studied in murine models in several ways. Firstly, the joint may be surgically destabilized such as in destabilization of medial meniscus (DMM) models, leading to altered weight-bearing and subsequently generalized OA changes, which includes cartilage loss. These models are reliable, reproducible, and have high disease penetrance. The time-course over which cartilage changes develop is delayed and therefore recapitulates the nature of human disease (Glasson et al., 2007). Cartilage can also be excised surgically through induction of an osteochondral defect. These defects may progress at different rates depending on the operative site (Haase et al., 2019), and so makes for difficulty in determining the optimal timing of EV treatment. In contrast, DMM models may progress to display cartilage loss at time points later than direct osteochondral injury and therefore may not benefit from EV treatment in the acute post-injury phase. The amount of lesion healing however appeared to be similar when comparing findings from Zhang et al. (2016) and Zhang et al. (2018), where the same treatment was given after DMM and direct osteochondral injury, respectively. Cartilage loss may also be induced using enzymes or chemicals that degrade the cartilage. Three studies induced chondral injury in murine models using collagenase and one study used monosodium iodoacetate (MIA). Chemical induction is less predictable and may also attenuate the effects of cell-based therapies mimicking its effects on tissue-native cells (Taghizadeh et al., 2018). One study focused on the temporomandibular joint (TMJ). As the pathoaetiology and epidemiology of these non-weight bearing joints typically differ, with radiographic TMJ cartilage loss often being asymptomatic (Schmitter et al., 2010), it may not be relevant to compare functional outcomes following treatment.

It is important to determine standardized methods of assessing outcomes relevant to cartilage repair. Several studies reported improvements in the macroscopic appearance of cartilage; while macroscopic appearance scoring is predictive of histological scoring (Goebel et al., 2017), it is unclear how this correlates with functional improvements. The three studies that used collagenase to induce cartilage loss, often termed collagenase induced OA (CIOA) models, assessed the highly clinically relevant outcomes of pain behavior, with both reporting reduction from early stages. It is difficult to draw conclusions from these results as cartilage loss and eventual OA in the murine model is typically late in onset and appears over 10 weeks following injury (Inglis et al., 2008). It is encouraging however, that all studies reported improved microscopic cartilage repair, with several studies employing various subjective quantitative scoring systems (Tao et al., 2017; Wang et al., 2017; Zhu Y. et al., 2017; Wu et al., 2019). It may be informative to conduct a pooled analysis of such outcomes, but it is unlikely to be meaningful in this study owing to the heterogeneity in scores used. Not all studies reported favorable outcomes when assessing markers of cartilage repair. Khatab et al. conducted an immunohistochemical analysis of iNOS and CD206 expression which are markers of inflammation and M2 macrophage, respectively (Fahy et al., 2014), and found no difference between the groups at the end of the study. Inflammation probably attenuates cartilage repair by interfering with chondrocyte activity and so could be of greater relevance in the acute stage (Tung et al., 2002). The majority of studies showed improved collagen immunostaining, and greater expression of chondrogenic genes in tissue samples. Several groups evaluated cell apoptosis as an outcome and found decreased expression of markers of apoptosis following treatment (Zhang et al., 2018; Jin et al., 2020). Indeed, chondrocyte apoptosis may be reflective of matrix depletion in the cartilage (Kim et al., 2001).

In addition to promoting chondrogenesis in chondrocytes, it is likely that EVs contribute to cartilage repair via effects on other cell types. Macrophages are postulated to be a key target for MSC-EVs, with evidence suggesting that EVs cause an M2 phenotype polarization that promotes resolution of inflammation and so promotes cartilage repair (Chen et al., 2019). This notion is supported by the fact that macrophages pre-conditioned with MSC-EVs, termed EV-educated macrophages (EEMs) support tendon healing *in vivo* to a greater extent than treatment with MSC-EVs alone (Chamberlain et al., 2019). Others suggest that MSC-EV secretome actually augments the immunomodulatory effects of MSCs via autocrine action. It appears that IL-1β-pretreated MSCs induce macrophages into an anti-inflammatory phenotype only when in the presence of EVs containing miR-146a (Song et al., 2017). Transplantation of circulating EVs from septic mice however, appear to encourage neutrophil migration and macrophage inflammation; this is attributed to certain miRNAs including miR-126-3p, miR-222-3p, and miR-181a-5p, suggesting that EV-dependent modulation of inflammation is content and context dependent (Xu et al., 2018). Further to this, caution should be exercised with MSC-EV transplantation in certain patient cohorts, as MSC-EV have been shown to attenuate the ability of macrophages to suppress cancer cells, and in doing so promotes tumorigenicity (Ren et al., 2019).

## Conclusion

Due to the plethora of pathways through which MSC-EVs can promote cartilage repair, a key step to studying their effects in animal models is to establish the roles of the different bioactive components within EVs. Similarly, outcome measures utilized in studies should complement this. We recommend that cartilage appearance and chondrogenic gene expression should be primary outcomes in addition to quantifiable and clinically relevant functional outcomes such as pain reduction or animal gait analysis. Likewise, it would be beneficial to establish a link between functional and histological outcomes, as the value of assessing histology may otherwise be minimal. In order to establish the optimal way to deliver clinical benefit using MSC-EVs, the most efficient MSC cell source, methodology of cell culture and EV purification should be investigated for the purposes of cartilage repair. Finally, randomized studies in animal models that recapitulates the human disease will be necessary in order to establish a dose-response relationship and therefore clinical safety before we proceed to human trials.

## Data Availability Statement

The original contributions presented in the study are included in the article/supplementary materials, further inquiries can be directed to the corresponding author/s.

## Author Contributions

KT and WK conceptualized the review manuscript. KT wrote the manuscript under the supervision of FH and WK. KT and AK assembled study data and conducted literature searches. KR and CM conducted data analysis and risk of bias analysis. All authors contributed to editing and approving the manuscript.

## Conflict of Interest

The authors declare that the research was conducted in the absence of any commercial or financial relationships that could be construed as a potential conflict of interest.
